# Acute Graft-vs.-Host Disease-Associated Endothelial Activation *in vitro* Is Prevented by Defibrotide

**DOI:** 10.3389/fimmu.2019.02339

**Published:** 2019-10-09

**Authors:** Julia Martinez-Sanchez, Hannah Hamelmann, Marta Palomo, Enrique Mir, Ana Belen Moreno-Castaño, Sergi Torramade, Montserrat Rovira, Ginés Escolar, Steffen Cordes, Martina Kalupa, Sarah Mertlitz, Katarina Riesner, Enric Carreras, Olaf Penack, Maribel Diaz-Ricart

**Affiliations:** ^1^Josep Carreras Leukaemia Research Institute, Hospital Clinic, University of Barcelona, Barcelona, Spain; ^2^Department of Hematopathology, Biomedical Diagnosis Center (CDB), Institute of Biomedical Research August Pi i Sunyer (IDIBAPS), Hospital Clinic of Barcelona, University of Barcelona, Barcelona, Spain; ^3^Barcelona Endothelium Team, Josep Carreras Leukaemia Research Institute, Barcelona, Spain; ^4^Hematology, Oncology and Tumor Immunology Department, Charité Universitätsmedizin Berlin, Berlin, Germany; ^5^Berlin-Brandenburg Center for Regenerative Therapies, Charité Medical University of Berlin, Berlin, Germany; ^6^Department of Hematology and Oncology, Berlin Institute of Health, Berlin, Germany; ^7^Stem Cell Transplantation Unit, IDIBAPS, Hospital Clinic, University of Barcelona, Barcelona, Spain

**Keywords:** endothelial dysfunction, angiogenesis, acute GVHD, hematopoietic cell transplantation, defibrotide

## Abstract

Angiogenesis and endothelial activation and dysfunction have been associated with acute graft-vs.-host disease (aGVHD), pointing to the endothelium as a potential target for pharmacological intervention. Defibrotide (DF) is a drug with an endothelium-protective effect that has been approved for the treatment of veno-occlusive disease/sinusoidal obstruction syndrome after allogeneic hematopoietic cell transplantation. Clinical data suggest that DF also reduces the incidence of aGVHD; however, the mechanisms of DF-mediated aGVHD regulation have not been examined. To investigate possible DF-mediated prophylactic and therapeutic mechanisms in aGVHD, we performed *in vitro* studies using endothelial cell (EC) lines. We found that DF significantly and dose-dependently suppressed EC proliferation and notably reduced their ability to form vascular tubes in Matrigel. To explore whether DF administered prophylactically or therapeutically has a significant effect on aGVHD endothelial dysfunction, ECs were exposed to media containing sera from patients with aGVHD (*n* = 22) in the absence or presence of DF and from patients that did not develop aGVHD (*n* = 13). ECs upregulated adhesion molecules (vascular cell adhesion molecule 1, intercellular adhesion molecule 1), the adherence junction protein VE-cadherin, von Willebrand factor (VWF), and Akt phosphorylation in response to aGVHD sera. These responses were suppressed upon treatment with DF. In summary, DF inhibits vascular angiogenesis and endothelial activation induced by sera from aGVHD patients. Our results support the view that DF has notable positive effects on endothelial biology during aGVHD.

## Introduction

Hematopoietic cell transplantation (HCT) has demonstrated to be an effective treatment for various hematological and non-hematological diseases. Allogeneic HCT (allo-HCT) is associated with various early complications, among which acute graft-vs.-host disease (aGVHD) is the major cause of morbidity and mortality after this treatment (1–5). aGVHD is characterized by tissue and organ injury mainly affecting the liver, gut, and skin (1). First-line treatment for patients undergoing aGVHD is limited to corticosteroid therapy and immunosuppressive drugs, such as calcineurin or mTOR inhibitors (1, 6, 7). Although aGVHD progression can be partly or completely reverted through the effects of these therapies on immune cells, they have a detrimental impact on the endothelium (8).

The endothelial cells (ECs) that cover the vascular tree are directly exposed to all the damaging factors occurring during the onset and development of aGVHD. Our group has previously demonstrated that allo-HCT is associated with endothelial dysfunction (9, 10) and that endothelial damage is aggravated with the development of aGVHD. Humoral factors in the sera of allo-HCT recipients developing aGVHD induced a marked proinflammatory and prothrombotic state in cultured ECs (11). Plasma levels of von Willebrand factor (VWF) and tumor necrosis factor receptor 1 were higher in patients developing aGVHD than in allo-HCT recipients not developing this complication. Other endothelial damage markers, including angiopoietin-2, were increased even in pretransplant patients who later developed aGVHD, corroborating that the endothelium plays a role in the pathogenesis of aGVHD (12). In addition, it has been considered that angiogenesis, the formation of new blood vessels, might play a major role in aGVHD development. It has been demonstrated that, in the early inflammatory phase of GVHD, there is a noticeable neovascularization that facilitates the migration of inflammatory cells to target organs (13). Together, these findings highlight the endothelium as a potential target for new therapies to prevent and treat aGVHD (11, 14, 15).

Defibrotide (DF), an endothelial-protective drug (16), has demonstrated to be effective in the prevention and treatment of veno-occlusive disease/sinusoidal obstruction syndrome (VOD/SOS), an early complication of HCT proven to be of endothelial origin (17). Our previous studies demonstrated the *in vitro* preventive effect of DF on the development of endothelial damage in association with HCT (18) and the mechanisms of action involved (19). DF, designated as an orphan drug for the prevention of aGVHD, is currently being evaluated for GVHD prevention in a clinical trial in adults (20).

The effect of DF has never been evaluated in an *in vitro* aGVHD-related endothelial damage scenario. The present study aimed to explore the effect of DF on endothelial cell proliferation and tube formation and its potential as a prophylactic or/and therapeutic agent for aGVHD-associated endothelial damage.

## Materials and Methods

### Endothelial Cell Culture

Primary human umbilical vein endothelial cells (HUVEC) were used to assess DF effect in proliferation and tube formation assays. Human microvascular endothelial cells (HMEC-1) (ATCC, Manassas, VA, USA) were used to investigate DF effect on a GVHD-endothelial dysfunction *in vitro* model. HUVEC were cultured in endothelial basal medium 2 (Lonza, Walkersville, MD, USA) supplemented with endothelial cell growth medium SingleQuots™ (EGM-2) (Lonza), human epidermal growth factor, hydrocortisone, GA-1000 (gentamicin, amphotericin-B), 2% fetal bovine serum, vascular endothelial growth factor, human basic fibroblast growth factor, R3 insulin growth factor-1, ascorbic acid, and heparin. HMEC-1 were grown in MCDB131 (Gibco-BRL, Madrid, Spain) medium supplemented with 15% fetal bovine serum, 4% l-glutamine, 1% penicillin-streptomycin (Gibco-BRL), 1 μg/ml hydrocortisone (Sigma-Aldrich, Madrid, Spain), and 10 ng/ml epidermal growth factor (BD Biosciences, Erembodegem, Belgium). Both HUVEC and HMEC-1 were cultured under humidified conditions at 37°C and in the presence of 5% CO_2_.

### MTT Proliferation Assay

The proliferation rate of cells was assessed by the 3-[4,5-dimethylthiazole-2-yl]-2,5-diphenyltetrazolium bromide (MTT) assay using the MTT Cell Proliferation Assay Kit (ATCC). HUVEC were seeded in three 96-well plates (Sarstedt, Nümbrecht, Germany) at 1–2 × 10^4^ cells/well in 100 μl of EGM-2. DF was added at 10, 100, or 500 μg/ml. The first two concentrations (10 and 100 μg/ml) were selected due to their clinical relevance (21, 22) and the last one (500 μg/ml) to analyze if maximum concentrations, not expected in the clinical routine, have an additional effect. Then, the plates were incubated at 37°C for 24 h (D + 1), 48 h (D + 2), or 72 h (D + 3), and 10 μl of MTT reagent was added to each well. After 3 h, 100 μl of Detergent Reagent was added to each well, and the plates were incubated in the dark (3–4 h or overnight) until the precipitated formazan crystals were dissolved. The absorbance at 570 nm was measured using a SpectraMax Plus 384 mL plate reader (Molecular Devices, San Francisco, CA, USA). The mean value from quadruplicate readings was determined.

### Tube Formation Assay

The angiogenic activity of cells was determined by a tube formation assay. Forty microliters of defrosted Matrigel® (Corning, Corning, NY, USA) was pipetted into the wells of a 96-well plate (Sarstedt), using a cut 10- or 100-μl pipette tip. The plate was left to stand for 30–45 min until the Matrigel was solidified. HUVEC (1 × 10^4^ cells) in 100 μL of medium containing DF at different concentrations were added to each well. Cells incubated in EGM-2 and endothelial basal medium 2 medium were used as a positive and negative control, respectively. Tube formation was evaluated every hour, and after 6 h, micrographs were taken of three to five random fields per well. The micrographs were analyzed using the Angiogenesis Analyzer software developed by Gilles Carpentier (http://image.bio.methods.free.fr/ImageJ/?Angiogenesis-Analyzer-for-ImageJ&lang=en) in ImageJ Fiji (Bethesda, Rockville, MD, USA) (23). The following features were assessed: number of junctions, number of master junctions, number of segments, number of master segments, number of meshes, mean mesh size, and total meshes area. The mean value from quadruplicate readings from two independent experiments was determined for each parameter. Student's unpaired *t*-test was applied for the statistical analysis of all data using the statistics program GraphPad Prism, version 4.02 (GraphPad Software Inc., La Jolla, CA, USA). The values were adjusted to the positive control and shown as percentage; the value of the positive control was adjusted to 100%. Values were shown as mean ± standard error of the mean (SEM).

### Exposure of Endothelial Cells in Culture to aGVHD Media

The effect of DF on aGVHD was assessed *in vitro*. HMEC-1 (24) were incubated in growth medium supplemented with 20% of serum pools, each pool containing combinations of sera from five different patients. Serum samples were collected from patients with aGVHD on the day of diagnosis. For experimental treatments, DF (100 μg/ml) was added in two modalities: (i) prophylaxis plus treatment (DF-PT), in which ECs were incubated with DF 24 h before exposure to aGVHD medium, and then fresh DF was added with medium replacement every 24 h, and (ii) therapeutic (DF-T), in which ECs were exposed to aGVHD medium, and DF was added 24 h later and at growth medium replacement every 24 h. The control group consisted of serum samples from allo-HCT recipients that did not develop aGVHD (NoGVHD).

### Patients and Specimen Collection

Patients undergoing allo-HCT at the Hospital Clinic of Barcelona were included consecutively between 2008 and 2010. The experimental group consisted of patients who developed aGVHD grade II–IV within the first 3 months post allo-HCT (aGVHD group, *n* = 22). The control group consisted of 13 patients without aGVHD (NoGVHD group). Blood samples were collected on the day of aGVHD diagnosis (before any change in treatment) for the aGVHD group and on day 21 post allo-HCT for the NoGVHD group. Patients with other early complications associated with allo-HCT such as VOD/SOS, thrombotic microangiopathy, capillary leak syndrome, or engraftment syndrome were excluded for this study. Serum was obtained by centrifugation at 2,500 rpm for 15 min and was stored at −80°C. Patient characteristics are provided in Table 1.

**Table 1:** Characteristics of patients with or without acute GVHD.

**Groups**	**GVHD**	**NoGVHD**
**Patients**		
Number (% of the whole group)	22 (63)	13 (27)
**Gender**		
Male/female	11/11	10/3
**Diagnostic**		
ALL	3	3
AML	8	5
NHL/HL	6	3
PCD	2	1
CLL/PLL	3	1
**Donor type**		
Sibling (% by group)	8 (36)	11 (85)
Unrelated (% by group)	14 (64)	2 (15)
**Conditioning**		
MAC (% by group)	12 (54)	9 (69)
RIC (% by group)	10 (46)	4 (31)
**GVHD Prophylaxis**		
CyA	2	–
CyA/MTX	10	4
CyA/MMF	9	8^*^
Tacro/Rapa	1	1
**Acute GVHD II–IV**		
Onset day, median (range)	16 (7–28)	NA
**Chronic GVHD**		
>at day+180 (% by group)	10 (78)	6 (46)

* *One case with CD34^+^ positive selection*.

*AML, acute myeloid leukemia; ALL, acute lymphoblastic leukemia; NHL, non-Hodgkin's lymphoma; HL, Hodgkin's lymphoma; PCD, plasma cell disease; CLL, chronic lymphocytic leukemia; PLL, prolymphocytic leukemia; MAC, myeloablative conditioning; RIC, reduced intensity conditioning; CyA, cyclosporine; MTX, methotrexate; MMF, mycophenolate mofetil; Rapa, rapamicine; Tacro, tacrolimus*.

### Immunofluorescence Analysis

The expression of the adhesion molecules intercellular adhesion molecule 1 (ICAM-1) and vascular cell adhesion molecule 1 (VCAM-1) on the cell surface, VE-cadherin at cell junctions, and VWF in the extracellular matrix (ECM) was assessed by immunofluorescence. HMEC-1 monolayers were incubated in medium containing 20% pooled serum from the aGVHD group, in the absence or presence of DF (DF-PT or DF-T), and from NoGVHD group in eight-well microplates (Nunklabtech, Madrid, Spain) for 48 h. ECs were fixed with 4% paraformaldehyde and incubated with specific monoclonal antibodies to VCAM-1 (MA5-11447, 1/10; Invitrogen, Carlsbad, CA, USA) and ICAM-1 (sc-107, 1/50; SantaCruz Biotech, Dallas, TX, USA) or permeabilized (0.05% Triton X-100, 3 min at room temperature) and then incubated with a specific monoclonal antibody to VE-cadherin (ab33168, 1/1,000; Abcam, Cambridge, UK) or a specific polyclonal antibody to VWF (A0082, 1/2,000; Dako/Agilent, Santa Clara, CA, USA), at room temperature for 1 h. Then, the cells were incubated with secondary antibodies conjugated with Alexa Fluor 488 or 594 (Molecular Probes, Eugene, OR, USA) at room temperature for 1 h. Cell monolayers were evaluated using a DM4000 B epifluorescence equipped microscope and images captured through a video camera (Leica, Barcelona, Spain). Analysis of fluorescence was carried out using ImageJ Fiji software in 20 fields per coverslip, by independent technicians blinded to the treatments.

### Reactivity of the ECM Generated by HMEC-1 Exposed to Study Conditions

ECs were cultured for 7 days on 18 × 18 mm^2^ coverslips in six-well plates in medium supplemented with 20% serum pools. The monolayers were exposed to 9% ethyleneglycoltetraacetic acid at 37°C for 1 h to remove ECs. To evaluate the reactivity of the ECM toward circulating platelets, the ECM-coated coverslips were perfused with citrated whole blood from healthy donors in a parallel-plate perfusion chamber (shear rate of 800/s, 5 min). After perfusion, the coverslips were fixed with 0.5% glutaraldehyde at 4°C and stained with 0.02% toluidine blue. Images were captured using the DM4000 B epifluorescence microscope. The surface covered by platelets (%) was quantified *en face* in 20 fields per well using ImageJ Fiji by independent technicians blinded to the treatments.

### Western Blotting Analysis of Phosphorylated Intracellular Proteins

Confluent EC monolayers, after starving with medium containing 2% serum for 24 h, were exposed to the serum pools under study with or without DF for 15 min. The ECs were lysed with Laemmli's buffer (68.5 mM tris[hydroxymethyl]aminomethane-HCl, 2% sodium dodecyl sulfate, 10% glycerol, 5 mM β-mercaptoethanol, and 0.003% bromophenol blue) containing 2 mM sodium orthovanadate, and 0.625 mg/ml *N*-ethylmaleimide. Proteins were resolved by 10% sodium dodecyl sulfate-polyacrylamide gel electrophoresis and transferred to nitrocellulose membranes (BioRad, Hercules, Canada), which were probed with specific antibodies to phosphorylated and unphosphorylated Akt, p38 MAPK, Erk1/2, and SAPK/JNK (Cell Signaling Technology, Danvers, MA, USA). Activation of proteins induced by NoGVHD sera was used as control.

### Statistical Analysis

Data are reported as the mean ± SEM. Data from aGVHD and NoGVHD exposure experiments following a normal distribution were analyzed by paired *t*-tests. Data from proliferation and tube formation experiments were analyzed by unpaired *t*-tests. All statistical analyses were conducted using GraphPad Prism, version 4.02. *P* < 0.05 was considered significant.

### Ethical Aspects

Informed consent for blood utilization was obtained from all patients. The study was approved by the Ethics committee of the Hospital Clinic of Barcelona (CEIC 2008/3353) and was carried out in accordance with the principles of the Declaration of Helsinki.

## Results

### DF Suppresses EC Proliferation and Tube Formation

**Figure 1:**
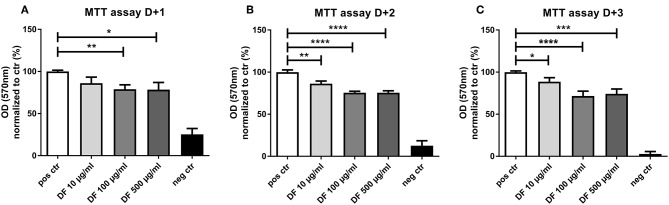
Defibrotide (DF) suppresses endothelial cell (EC) proliferation. An 3-[4,5-dimethylthiazole-2-yl]-2,5-diphenyltetrazolium bromide (MTT) assay was used to quantify the effect of DF (10, 100, or 500 μg/ml) for **(A)** 24 h (D + 1), **(B)** 48 h (D + 2), or **(C)** 72 h (D + 3) on EC proliferation. Bar diagrams represent the optical density (OD) normalized to control data (%) expressed as the mean ± SEM of three experiments. *P < 0.05, **P < 0.01, ***P < 0.001, ****P < 0.0001.

As aGVHD has been associated with angiogenesis, we investigated the effect of DF on the angiogenic potential of ECs. First, we assessed the impact of DF on EC proliferation by MTT assay. DF reduced EC proliferation in a time- and dose-dependent manner (Figure 1). Of note, even at the lowest concentration of 10 μg/ml, DF significantly inhibited EC proliferation. The reduction in the proliferation was more notable at 100 μg/ml (Figures 1B,C corresponding to MTT assay day + 2 and day + 3), and no differences were observed between 100 and 500 μg/ml concentrations.

**Figure 2:**
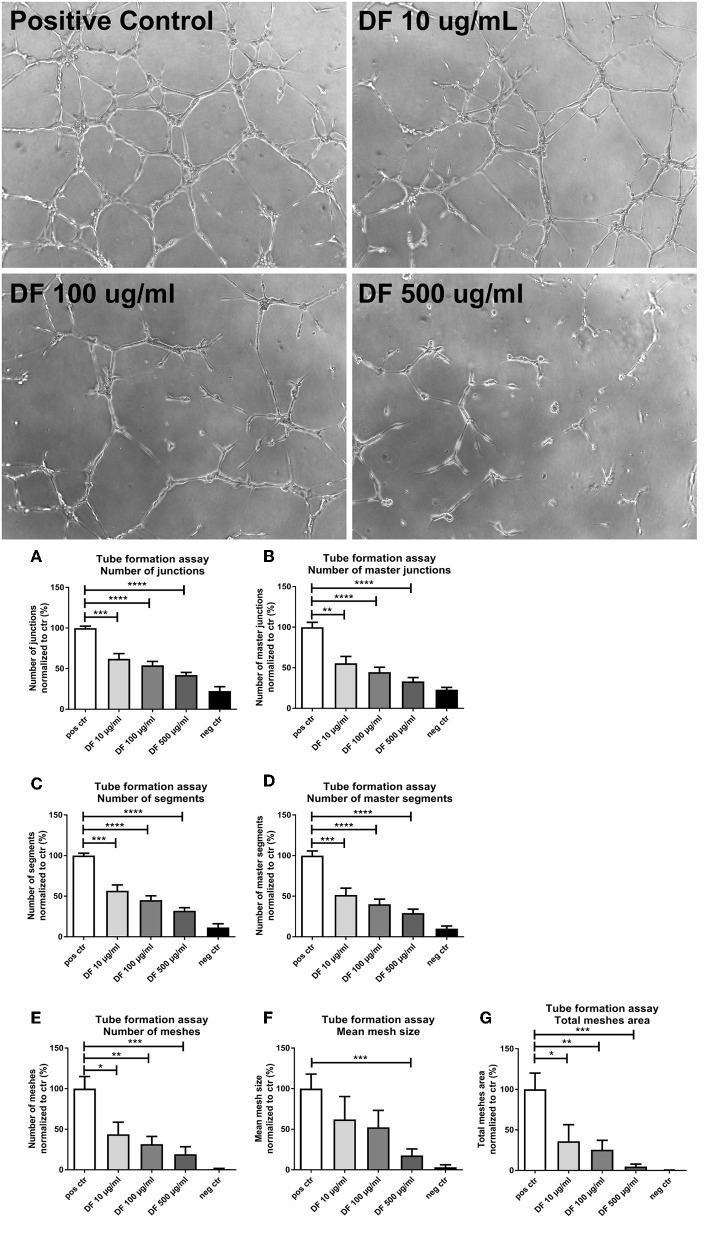
Defibrotide (DF) suppresses endothelial cell (EC) tube formation. Representative images of angiogenic activity of ECs by a tube formation assay incubated with DF at different concentrations. Bar diagrams represent different characteristics measured during tube formation: numbers of **(A)** junctions and **(B)** master junctions, numbers of **(C)** segments and **(D)** master segments, and **(E)** number, **(F)** size, and **(G)** area of meshes, as indicated. Data are expressed as the mean ± SEM of three experiments. *P < 0.05, **P < 0.01, ***P < 0.001, ****P < 0.0001.

Next, we assessed the impact of DF on the angiogenic activity of ECs by a tube formation assay. Cells were incubated in the presence of 10, 100, or 500 μg/ml DF in a 96-well plate coated with Matrigel and analyzed after 6 h (micrographs in Figure 2). DF significantly suppressed tube formation in a dose-dependent manner for all features assessed: junctions (Figure 2A), master junctions (Figure 2B), segments (Figure 2C), master segments (Figure 2D), meshes (Figure 2E), mean mesh size (Figure 2F), and total meshes area (Figure 2G). Thus, DF significantly suppressed EC proliferation and tube formation *in vitro*.

### DF Prevents aGVHD Serum-Induced Expression of Adhesion Molecules and VE-Cadherin on ECs

**Figure 3:**
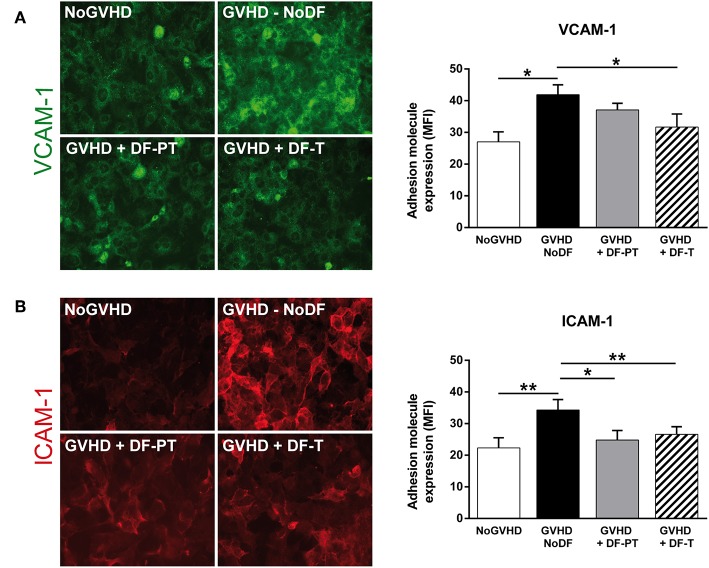
Defibrotide (DF) has an anti-inflammatory effect on endothelial cells (ECs) activated and injured after exposure to acute graft-vs.-host disease (aGVHD) serum. Representative images of **(A)** vascular cell adhesion molecule 1 (VCAM-1) and **(B)** intercellular adhesion molecule 1 (ICAM-1) expression on EC surfaces in response to aGVHD serum collected on the day of onset in the absence (GVHD-NoDF) or presence of DF (prophylaxis and treatment, GVHD + DF-PT; treatment, GVHD + DF-T). An image representative of results with NoGVHD medium is also included. Bar diagrams show the quantitative analysis of VCAM-1 and ICAM-1 for GVHD-NoDF (black bars), GVHD + DF-PT (gray bars), GVHD + DF-T (dashed bars), and NoGVHD (white bars). Values represent mean ± SEM of MFI from eight different experiments. *P < 0.05, **P < 0.01. Original magnification, 400× for all micrographs.

To analyze the effect of soluble factors present in aGVHD patient serum on ECs, HMEC-1 were cultured with pooled aGVHD or NoGVHD serum samples for 48 h, and the expression of surface proteins was analyzed by immunofluorescence. VCAM-1 and ICAM-1 levels were significantly increased in the presence of aGVHD serum compared to NoGVHD serum (Figure 3). The mean fluorescence intensity (MFI of eight different experiments) for VCAM-1 and ICAM-1 was 41.9 ± 3.1 and 34.3 ± 3.3, respectively, in the presence of aGVHD serum vs. 27 ± 3.2 and 22.3 ± 3.2, respectively, in NoGVHD (*P* < 0.05 and *P* < 0.01, respectively). To analyze whether DF can suppress the aGVHD-induced upregulation of adhesion molecule expression, we incubated ECs with aGVHD serum and DF in two regimens: (i) prophylaxis plus treatment (DF-PT), in which ECs were incubated with DF 24 h before exposure to aGVHD medium, and then fresh DF was added with medium replacement every 24 h; and (ii) therapeutic (DF-T), in which ECs were exposed to aGVHD medium, and DF was added 24 h later and at growth medium replacement every 24 h. Figure 3 shows that aGVHD serum-induced expression of VCAM-1 and ICAM-1 was significantly suppressed in the DF-PT group (to 37.1 ± 2.1 and 24.8 ± 3, respectively, *P* < 0.05) and the DF-T group (to 31.7 ± 4.1 and 26.6 ± 2.4, respectively, *P* < 0.01).

**Figure 4:**
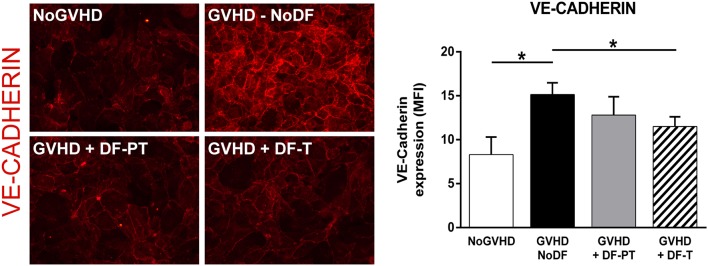
Defibrotide (DF) attenuates the increase in VE-cadherin expression on the surface of endothelial cells (ECs) exposed to acute graft-vs.-host disease serum. VE-cadherin staining in ECs exposed to aGVHD serum in the absence (GVHD-NoDF) or presence of DF. An image representative of results with NoGVHD medium is also included. Bar diagrams show the quantitative analysis of VE-cadherin expression for GVHD-NoDF (black bar), GVHD + DF-PT (gray bar), GVHD + DF-T (dashed bar), and NoGVHD (white bar). Values indicate the mean ± SEM of MFI from eight different experiments. *P < 0.05. Original magnification, 400× for all micrographs.

Next, we analyzed the expression of VE-cadherin, an adherence junction protein involved in angiogenesis and vascular integrity, on ECs exposed to aGVHD or NoGVHD serum. aGVHD serum enhanced VE-cadherin expression compared to NoGVHD serum (MFI of eight different experiments, 15.14 ± 1.34 vs. 8.3 ± 2, *P* < 0.05). DF suppressed the VE-cadherin upregulation in the prophylactic as well as the therapeutic setting (DF-PT, 12.8 ± 2.1; DF-T, 11.5 ± 1.1, *P* < 0.05) (Figure 4). Thus, DF reduced aGVHD serum-induced upregulation of adhesion molecule and VE-cadherin expression on ECs *in vitro*.

### DF Inhibits the Release of VWF From ECs to the ECM

**Figure 5:**
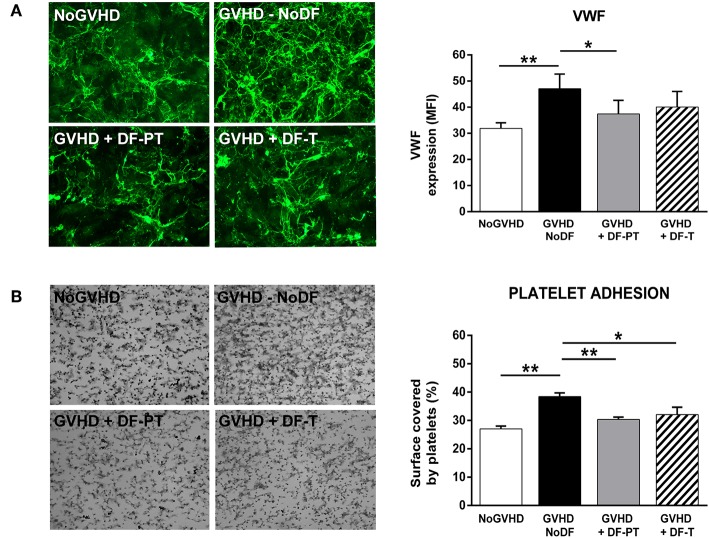
Defibrotide (DF) reduces von Willebrand factor (VWF) expression and platelet adhesion on extracellular matrix (ECM) produced by endothelial cells (ECs) exposed to aGVHD serum. **(A)** Micrographs showing expression of VWF on ECM produced by ECs exposed to aGVHD serum in the absence (GVHD-NoDF) or presence of DF (DF-PT and DF-T). An image representative of results with NoGVHD medium is also included. Bar diagrams show the quantitative analysis of VWF expression for GVHD-NoDF (black bar), GVHD + DF-PT (gray bar), GVHD + DF-T (dashed bar), and NoGVHD (white bar). Data are the mean ± SEM of MFI from eight different experiments. *P < 0.05. Original magnification, 400× for all micrographs. **(B)** Representative images showing platelet adhesion after exposing ECM produced under the indicated conditions to flowing blood (800/s, 5 min). Bar diagrams show the surface covered by platelets (%) for GVHD-NoDF (black bar) GVHD + DF-PT (gray bar), GVHD + DF-T (dashed bar), and NoGVHD (white bar). Data are the mean ± SEM of MFI from eight different experiments. *P < 0.05, **P < 0.001. Original magnification, 200× for all micrographs.

To investigate the potential antithrombotic effect of DF, either DF-PT or DF-T, in aGVHD endothelial damage, VWF expression in ECM generated by ECs grown in the presence of aGVHD or NoGVHD with or without DF was evaluated by immunofluorescence. aGVHD serum enhanced VWF expression compared to NoGVHD serum. The MFI of VWF, based on eight different experiments, was 47.1 ± 5.6 for aGVHD vs. 31.9 ± 2.1 for NoGVHD (*P* < 0.01). Both DF-PT and DF-T significantly suppressed VWF production, as indicated by MFIs of 37.4 ± 5.2 for DF-PT (*P* < 0.05) and 40.1 ± 5.9 for DF-T (Figure 5A).

### DF Reduces the Increased Reactivity of the ECM Toward Platelets Caused by Exposure to aGVHD Serum

The effects of aGVHD serum and DF on ECM thrombogenicity were studied under flow conditions. ECMs generated by ECs grown in the presence of aGVHD or NoGVHD serum for 7 days were exposed to circulating citrated blood from healthy donors, and the surface covered by platelets was determined. The covered surface (expressed in percent with respect to the total area of the field analyzed, mean ± SEM, *n* = 8) was higher in the aGVHD than in the NoGVHD group (38.4 ± 1.3 vs. 27 ± 1%, *P* < 0.01). DF-PT and DF-T significantly reduced surface coverage to 30.4 ± 0.8% (*P* < 0.001) and to 32.1 ± 2.6% (*P* < 0.05), respectively (Figure 5B).

### DF Suppresses the Activation of Akt in ECs Exposed to Serum From aGVHD Patients

**Figure 6:**
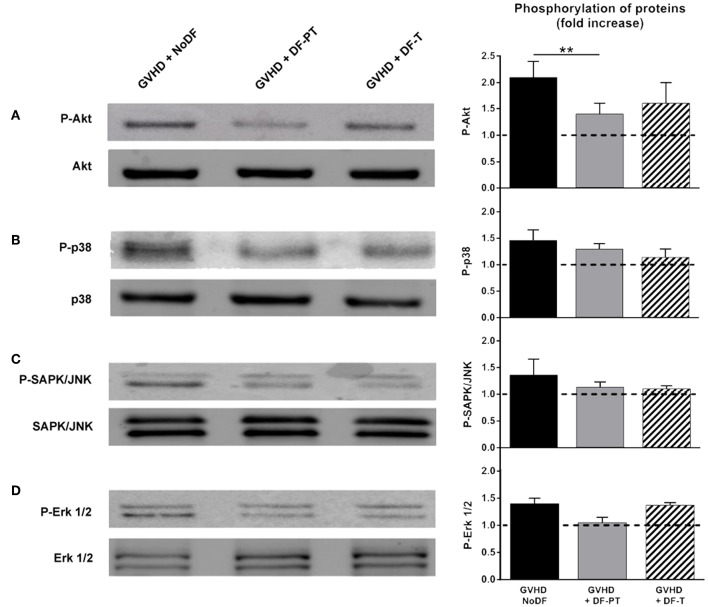
Defibrotide (DF) suppresses the activation of Akt in endothelial cells (ECs) exposed to serum from aGVHD patients. ECs were exposed for 15 min to aGVHD serum in the absence (GVHD-NoDF) or presence of DF (DF-PT and DF-T) to evaluate the effect of DF on the phosphorylation of **(A)** Akt (P-Akt), **(B)** p38 MAPK (P-p38), **(C)** SAPK/JNK (P-SAPK/JNK), and **(D)** Erk1/2 (P-Erk1/2) by Western blotting. Images are representative of four experiments. Bar diagrams represent the increases in phosphorylation with respect to the phosphorylation levels in cells exposed to NoGVHD serum. Data are the mean ± SEM from four different experiments **P < 0.01. Results corresponding to NoGVHD are represented by a dotted line.

The effects of aGVHD serum and DF on phosphorylation-mediated activation of Akt, p38 MAPK, SAPK/JNK, and Erk1/2 were investigated by Western blotting. Akt phosphorylation was significantly higher in cells exposed to aGVHD serum (fold increase of 2.1 ± 0.3 in aGVHD vs. NoGVHD, *P* < 0.01). DF-PT and DF-T significantly suppressed Akt phosphorylation in response to aGVHD serum to 1.4 ± 0.2 and 1.6 ± 0.4, respectively (Figure 6A). Similar trends were observed for p38, SAPK/JNK, and Erk1/2, with fold increases of 1.46 ± 0.2, 1.36 ± 0.3, and 1.4 ± 0.1 in aGVHD vs. NoGVHD, respectively. DF-PT reduced the activation of p38, SAPK/JNK, and Erk1/2 to 1.3 ± 0.1, 1.13 ± 0.1, and 1.05 ± 0.1, respectively, and DF-T to 1.14 ± 0.16, 1.1 ± 0.06, and 1.37 ± 0.05, respectively (Figures 6B–D). Differences between groups were only statistically significant for Akt.

## Discussion

We investigated the effect of DF on angiogenesis and its potential preventive and restorative effects on endothelial injury caused by aGVHD in an *in vitro* cell culture model. Experiments performed to explore the effect of DF on angiogenesis showed that this compound suppressed both proliferation and tube formation capability in ECs. In addition, DF prevented and partially reversed the endothelial inflammatory and prothrombotic phenotypes induced by aGVHD serum. Interestingly, our *in vitro* experiments demonstrated that DF is capable of restoring endothelial damage when administered therapeutically. Our results suggest a potential role of this drug as a prophylactic and therapeutic agent for endothelial dysfunction associated with aGVHD.

Owing to advances in the knowledge of aGVHD pathophysiology in the last decade, the vascular endothelium was identified as a new target for the prevention and treatment of this complication (6, 15, 25–28). In the initial phases of aGVHD development, angiogenesis precedes key processes, such as inflammatory cell infiltration in typical aGVHD target organs (29). However, blood vessels suffer fibrosis and rarefaction during later stages of aGVHD due to the attack of alloreactive donor T cells on the vasculature (14, 30). Inhibition of angiogenesis during the early phases of aGVHD may suppress the recruitment of infiltrating leukocytes and, consequently, inflammation, and thus may improve aGVHD (13, 29). Moreover, there is evidence for the association between angiogenesis and the expression of VE-cadherin, the main endothelial cell-to-cell junction transmembrane protein that functions in vascular morphogenesis and endothelial survival, on ECs (31, 32). In this regard, Medinger et al. (33) reported higher microvessel density and VE-cadherin expression in bone marrow sections from patients with aGVHD than from patients without aGVHD. In line herewith, we also found that VE-cadherin expression was elevated in ECs exposed to aGVHD serum. In addition, VE-cadherin was clearly downregulated by DF, mostly when ECs were incubated with DF after 24 h of exposure to aGVHD serum. The effect of DF on angiogenesis has been controversial. On the one hand, DF has been shown to bind to and protect basic fibroblast growth factor, a key angio-protective protein, thus promoting EC mitogenesis *in vitro* (34), and to stimulate hepatic sinusoidal EC angiogenesis in a dose-dependent manner (35). On the other hand, it has been demonstrated that DF directly interferes with EC migration and tube formation *in vitro* and in animal models (36). Our results regarding new vessel formation and VE-cadherin expression support the antiangiogenic role of this drug.

The current study also provided new evidence indicating that the treatment of ECs with DF is also effective in restoring the inflammatory phenotype induced by aGVHD. The exposure of ECs to pooled sera from aGVHD recipients induced increases in cell-surface expression of the adhesion receptors VCAM-1 and ICAM-1. DF had an anti-inflammatory effect as it suppressed the expression of these receptors upon therapeutic and prophylactic plus therapeutic administration. This strategy has already been explored in the context of aGVHD; studies in mouse models have shown that suppression of effector T cell recruitment by blocking chemokine receptors and their ligands might be an interesting approach to reducing GVHD (37–39).

ECs grown in the presence of sera from HCT recipients generate an ECM enriched with VWF and tissue factor, which are responsible for increased reactivity toward circulating platelets (9). This prothrombotic phenotype was even more notable in ECs exposed to sera from aGVHD patients than in ECs exposed to NoGVHD serum. DF was able to suppress this overproduction and/or release of VWF to the ECM. These results are in line with the antithrombotic–thrombolytic behavior of DF demonstrated in several experimental models (16) and with results from clinical trials that demonstrated that DF restores the thrombolytic–fibrinolytic balance in patients suffering from VOD/SOS (40). DF has been also proposed for the treatment of thrombosis associated with various pathologies, including cerebral malaria, in which DF treatment leads to the maintenance of thrombomodulin and the suppression of platelet aggregation (41).

Sera from aGVHD patients disrupted endothelial homeostasis and induced strong activation of the PI3K-Akt-mTOR stress-signaling pathway. DF suppressed phosphorylation-dependent Akt activation nearly to control levels. Akt is related to processes devoted to EC stabilization during a dysfunctional state (42, 43), and its dysregulation has been extensively studied in aGVHD, although not in endothelium. PI3K/Akt pathway inhibitors can interfere with T cell activation, and their therapeutic utility in aGVHD has been explored (44, 45). The endothelial damage caused by leukocyte transmigration implies the activation of the inflammatory protein p38, the apoptosis-related protein SAPK/JNK, and the cell proliferation, differentiation, and survival protein Erk1/2 (46). In the current study, all these proteins were consistently, to different extents, more strongly activated in cells exposed to aGVHD serum than in cells exposed to NoGVHD serum. DF prevented and reduced the activation of all these proteins.

The present study demonstrated that DF inhibits EC proliferation and tube formation. Considering that angiogenesis precedes the onset of aGVHD, our results suggest that DF might be useful for the prevention and treatment of aGVHD. In addition, DF ameliorates the dysfunctional endothelial phenotype caused by aGVHD serum by preventing inflammatory and prothrombotic cell reactions and suppresses the activation of the stress-related protein Akt. Interestingly, DF is able to restore established endothelial damage. The present study was performed *in vitro*, and more research is needed to corroborate our findings. However, our results warrant further study of the usefulness of DF for the prevention and treatment of aGVHD. Clinical trials on aGVHD prophylaxis by DF are currently going.

## Data Availability Statement

The raw data supporting the conclusions of this manuscript will be made available by the authors, without undue reservation, to any qualified researcher.

## Ethics Statement

This study was carried out in accordance with the recommendations of Ethics committee of the Hospital Clinic of Barcelona with written informed consent from all subjects. All subjects gave written informed consent in accordance with the Declaration of Helsinki. The protocol was approved by the Hospital Clinic of Barcelona (CEIC 2008/3353).

## Author Contributions

JM-S, EM, and HH designed and performed the experiments, analyzed the results, and prepared the images. JM-S and EM wrote the manuscript. MP, ST, and AM-C contributed to experimental design, image preparation, and reviewed the data and the manuscript. MR provided the samples and clinical patient data. MR, SC, MK, SM, KR, AM-C, ST, OP, and GE reviewed the data and the manuscript. EC, OP, and MD-R designed and supervised the project and reviewed the data and the manuscript. All authors read, edited, and approved the manuscript.

### Conflict of Interest

This research has been partially supported by Jazz Pharmaceuticals plc/Gentium Inc. MP declares conflict of interest with Jazz Pharmaceuticals plc/Gentium Inc. in the form of speaker's fee for symposiums. EC, OP, and MD-R received research funding and speaker's fees from Jazz Pharmaceuticals. The remaining authors declare that the research was conducted in the absence of any commercial or financial relationships that could be construed as a potential conflict of interest.

## Abbreviations

aGVHD: acute graft-vs.-host disease
allo-HCT: allogeneic hematopoietic cell transplantation
DF: defibrotide
DF-PT: defibrotide—prophylaxis plus treatment
DF-T: defibrotide—therapeutic
EC: endothelial cell
ECM: extracellular matrix
HCT: hematopoietic cell transplantation
HMEC-1: human microvascular endothelial cells
HUVEC: human umbilical vein endothelial cells
ICAM-1: intercellular adhesion molecule 1
MFI: mean fluorescence intensity
SEM: standard error of the mean
TNFR1: tumor necrosis factor receptor 1
VCAM-1: vascular cell adhesion molecule 1
VOD/SOS: veno-occlusive disease/sinusoidal obstruction syndrome
VWF: von Willebrand factor.
